# Cooling to Hypothermic Circulatory Arrest by Immersion vs. Cardiopulmonary Bypass (CPB): Worse Outcome After Rewarming in Immersion Cooled Pigs

**DOI:** 10.3389/fphys.2022.862729

**Published:** 2022-03-31

**Authors:** Ole Magnus Filseth, Stig Eggen Hermansen, Timofei Kondratiev, Gary C. Sieck, Torkjel Tveita

**Affiliations:** ^1^Anesthesia and Critical Care Research Group, Faculty of Health Sciences, Department of Clinical Medicine, UiT The Arctic University of Norway, Tromsø, Norway; ^2^Division of Surgical Medicine and Intensive Care, University Hospital of North Norway, Tromsø, Norway; ^3^Emergency Medical Services, University Hospital of North Norway, Tromsø, Norway; ^4^Cardiothoracic Research Group, Faculty of Health Sciences, Department of Clinical Medicine, UiT The Arctic University of Norway, Tromsø, Norway; ^5^Cardiothoracic and Respiratory Medicine, University Hospital of North Norway, Tromsø, Norway; ^6^Department of Physiology and Biomedical Engineering, Mayo Clinic College of Medicine & Science, Rochester, MN, United States

**Keywords:** hypothermia induced, hypothermia accidental, cardiopulmonary bypass, immersion cooling, rewarming, cardiac index

## Abstract

**Introduction:**

Cooling by cardiopulmonary bypass (CPB) to deep hypothermic cardiac arrest (HCA) for cardiac surgical interventions, followed by CPB-rewarming is performed on a routine basis with relatively low mortality. In contrast, victims of deep accidental hypothermia rewarmed with CPB generally have a much worse prognosis. Thus, we have developed an intact pig model to compare effects on perfusion pressures and global oxygen delivery (DO_2_) during immersion cooling versus cooling by CPB. Further, we compared the effects of CPB-rewarming between groups, to restitute cardiovascular function, brain blood flow, and brain metabolism.

**Materials and Methods:**

Total sixteen healthy, anesthetized juvenile (2–3 months) castrated male pigs were randomized in a prospective, open placebo-controlled experimental study to immersion cooling (IMM_*c*_, *n* = 8), or cooling by CPB (CPB_*c*_, *n* = 8). After 75 minutes of deep HCA in both groups, pigs were rewarmed by CPB. After weaning from CPB surviving animals were observed for 2 h before euthanasia.

**Results:**

Survival rates at 2 h after completed rewarming were 4 out of 8 in the IMM_*c*_ group, and 8 out of 8 in the CPB_*c*_ group. Compared with the CPB_*c*_-group, IMM_*c*_ animals showed significant reduction in DO_2_, mean arterial pressure (MAP), cerebral perfusion pressure, and blood flow during cooling below 25°C as well as after weaning from CPB after rewarming. After rewarming, brain blood flow returned to control in CPB_*c*_ animals only, and brain micro dialysate-data showed a significantly increase in the lactate/pyruvate ratio in IMM_*c*_ vs. CPB_*c*_ animals.

**Conclusion:**

Our data indicate that, although global O_2_ consumption was independent of DO_2_, regional ischemic damage may have taken place during cooling in the brain of IMM_*c*_ animals below 25°C. The need for prolonged extracorporeal membrane oxygenation (ECMO) should be considered in all victims of accidental hypothermic arrest that cannot be weaned from CPB immediately after rewarming.

## Introduction

Cooling by cardiopulmonary bypass (CPB) until deep hypothermic cardiac arrest (HCA) at 15–18°C to perform complex aortic surgery followed by rewarming has been proven to be relatively safe over the last 10–15 years. Even in patients with serious co-morbidity, it has been reported that the overall hospital mortality associated with CPB is only around 5% (Bakaeen et al., 2010; Fehrenbacher et al., 2010). These results contrast with the reported hospital mortality of 70–87% after extracorporeal rewarming of victims of accidental hypothermia (Kornberger and Mair, 1996; Farstad et al., 2001). In many of these cases, hypothermia is secondary and preceded by asphyxia, as during submersion (drowning) (Farstad et al., 2001), or by burying in avalanches (Boyd et al., 2010). Successful CPB rewarming from accidental circulatory arrest at low body core temperatures has proven to be demanding, even in fit young survivors. Cardiopulmonary failure as well as renal insufficiency occurred early during intensive care treatment, irrespective if circulatory arrest was caused by primary hypothermia, and/or the interval between cardiac arrest and qualified cardiopulmonary resuscitation was relatively short (Gilbert et al., 2000; Oberhammer et al., 2008).

Fortunately, accidental hypothermia has received a lot of attention over the past 15 years (Paal et al., 2013), which has contributed to the creation of new treatment algorithms (Paal et al., 2016, 2022) that can be used in trauma centers. Progress has also been made after introduction of new techniques (Winkler et al., 2016), like extracorporeal membrane oxygenation (ECMO), for rewarming accidental hypothermia victims in cardiac arrest. The use of ECMO may be associated with higher survival rates and more favorable neurological outcomes in these patients than after rewarming using traditional CPB (Ruttmann et al., 2007; Morita et al., 2011).

Experimental data support the notion that the process of accidental surface cooling is more detrimental to the organism than the controlled clinical cooling by invasive or non-invasive methods. One apparent difference is the lack of sedation in accidental hypothermia. Experiments on newborn piglets demonstrated that the neuroprotective effect of mild hypothermia (35°C) after hypoxic brain damage was lost, if cooling was performed without deep sedation (Thoresen et al., 2001). Clinically it has been observed that surviving victims of accidental hypothermia influenced by sedative drugs or ethanol tolerated hypothermia and rewarming are better than victims unaffected by these substances (Locher et al., 1991; Vassal et al., 2001). If surface cooling in an anesthetized animal proceeds to deep hypothermia, preclinical studies have demonstrated that a hypothermia-induced, non-ischemic heart failure evolves, depending on the level of hypothermia and exposure time (Fedor et al., 1958; Popovic and Kent, 1965; Kondratiev et al., 2008; Filseth et al., 2010).

On the other hand, cooling by CPB and maintained CPB at low temperatures also have negative side effects. Hypothermic CPB is pro-inflammatory (Tassani et al., 2002), leads to post-hypothermic impaired cardiac ventricle function (Schultz et al., 2006), and invariably causes increased extravasation of fluid and general edema (Heltne et al., 2001; Farstad et al., 2003).

The goal of the present study was to isolate the effects of immersion cooling *per se* from cooling by CPB by keeping important factors like deep sedation and controlled ventilation similar in both groups. Thus, the immersion cooling protocol in the present study differed from the situation in accidental hypothermia, but we hypothesized that there would be fundamental differences in physiological responses to the two cooling techniques that would have translational therapeutic value for victims of accidental HCA.

## Materials and Methods

There are several alternative nomenclatures for hypothermia (Popovic and Popovic, 1974; Moss, 1986). We have chosen the definition by the American Heart Association (Vanden Hoek et al., 2010), which designates 30°C as a “watershed” marker between moderate (30–34°C) and potentially life-threatening (below 30°C) hypothermia, which is referred to as deep hypothermia if applied clinically and severe, if caused accidentally.

### Animals and Overall Experimental Protocol

**FIGURE 1:**
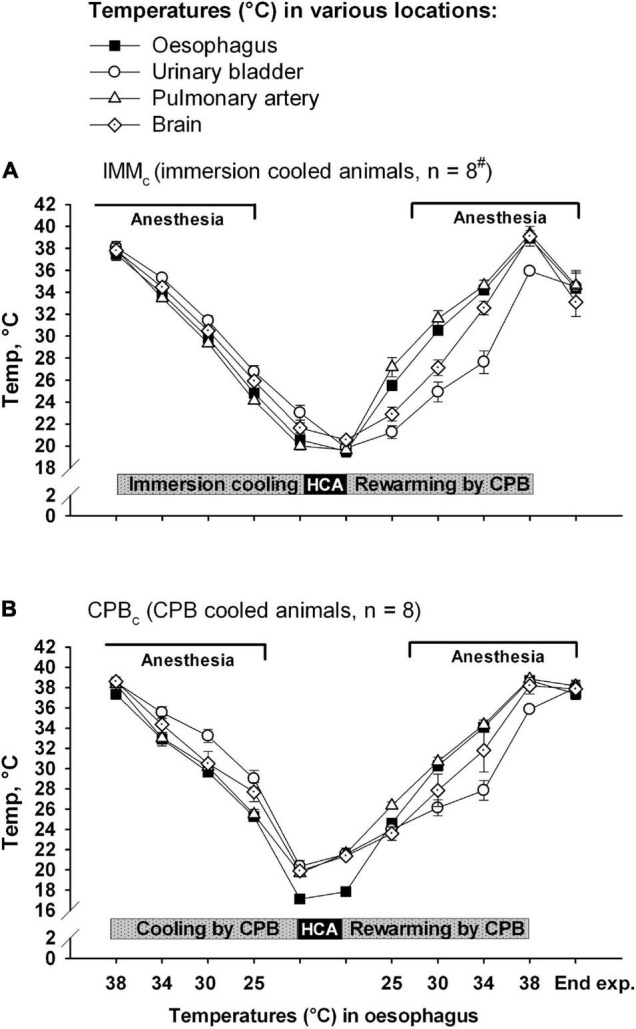
Temperatures at various body locations. **(A)** Immersion cooled animals (IMM_*c*_ group), and **(B)** in animals cooled by CPB (CPB_*c*_ group). Data presented as mean and SEM. ^#^n was reduced to 4 animals at the end of experiments (End exp.) in IMM_*c*_ group.

**FIGURE 2:**
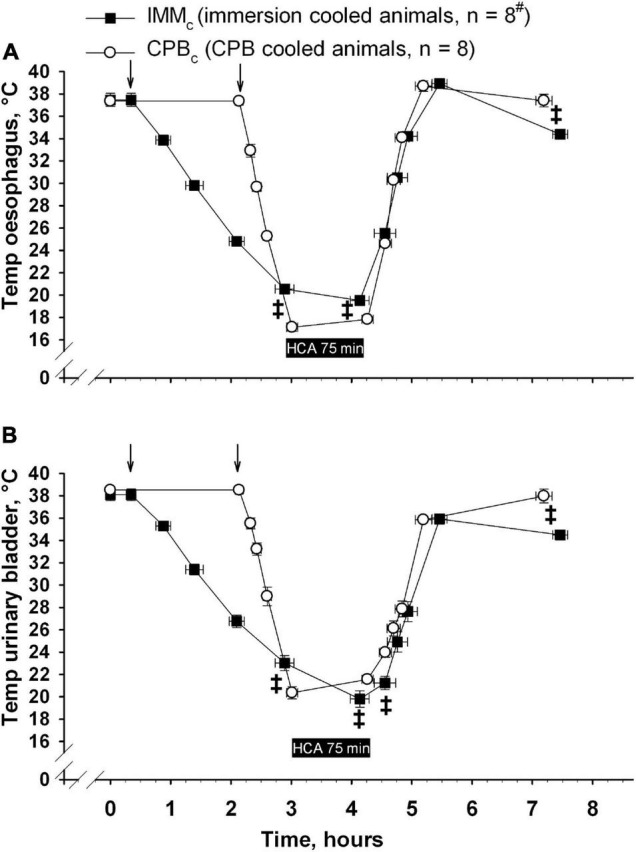
Time-dependent changes in temperature in esophagus **(A)** and urinary bladder **(B)**. ↓ denotes start of cooling in either group. Data presented as mean and SEM. ^‡^Statistically significant difference between groups (*p* < 0.05). ^#^n was reduced to 4 animals at end of the experiments (End exp.) in IMM_*c*_ group.

Animals received human care in accordance with The Norwegian Animal Welfare Act and the study (reference number: 37/04) was approved by The Norwegian Animal Research Authority (Forsøksdyrutvalget). Total sixteen castrated male juvenile pigs (24–37 kg, 2–3 months of age) from a hybrid crossing of native Norwegian (norsk landsvin) and British Yorkshire breeds were placed in pens for 2–5 days after arrival to the laboratory animal unit. They were fed twice daily and had free access to water at all the times. The animals were anesthetized and randomized to immersion cooling (IMM_*c*_, *n* = 8) or cooling by CPB (CPB_*c*_, *n* = 8) groups. Cooling continued until hypothermic circulatory arrest (HCA). After 75 minutes of HCA animals in both the groups were rewarmed by CPB. Following weaning from CPB surviving animals were observed for 2 h before euthanasia by an intravenous bolus of 20 mmol potassium chloride during ongoing anesthesia. The overall protocol layout is presented in Figures 1, 2. Figure 1 shows temperature variations in different body locations, time-points for sampling, administration of anesthesia, and cooling/rewarming methods in IMM_*c*_ (Figure 1A) and CPB_*c*_ (Figure 1B) groups. Figure 2 shows the time-course of experiments and compares time-dependent temperatures in esophagus (Figure 2A) and urinary bladder (Figure 2B) in the two groups.

### Anesthesia and Instrumentation

The animals were fasted overnight before premedication was induced in the pen by a bolus intramuscular injection of ketamine hydrochloride 20 mg/kg, midazolam 25 mg, and atropine 1.0 mg. After weighing and transferring the animals to the operation theater, anesthesia was induced by a bolus infusion of intravenous fentanyl 10 μg/kg, and pentobarbital-sodium 10 mg/kg. Following tracheostomy, the right external jugular vein was catheterized for infusion of fentanyl 20 μg/kg/h, pentobarbital-sodium 4 mg/kg/h, and midazolam 0.3 mg/kg/h together with Ringer‘s acetate 9 ml/kg/h. This infusion was maintained throughout the experiment, except for the period when brain temperature was below 25°C.

Animals were ventilated with a PEEP of 4 cm H_2_O (Siemens Servo 900 D, Solna, Sweden). The fractional concentration of O_2_ (FiO_2_) was adjusted to maintain PaO_2_ > 10 kPa, and alveolar ventilation was adjusted to keep PaCO_2_ at 4.5–6.0 kPa, uncorrected for temperature (alpha-stat management).

An arterial catheter was positioned into the left femoral artery for monitoring mean arterial pressure (MAP) and blood sampling. A 5F thermodilution catheter (Edwards Lifesciences, Irvine, California, United States) was advanced *via* the right external jugular vein for monitoring of central venous pressure (CVP), pulmonary artery pressure (PAP), blood temperature; cardiac output (CO), and central venous saturation. A 6F pigtail high-volume-flush catheter (Cordis, Miami, Florida, United States) was positioned into the left ventricle of the heart through the left carotid artery for monitoring left ventricular pressure. At the end of experiments, the animals were re-weighed.

### Recording and Calculation of Hemodynamic Variables

Electrocardiogram (ECG) (from standard leads), heart rate (HR), CVP, MAP, and PAP were continuously displayed on a data monitor and intermittently recorded using the software package LabVIEW™ v.6.0 (National Instruments, Austin, TX, United States). In addition, left ventricular end diastolic pressure (LVEDP) was recorded and maximum and minimum values of the first derivate of ventricular pressure over time (dP/dt_*max*_ and dP/dt_*min*_) were calculated by the LabVIEW software.

Cardiac output (CO) was measured by a thermodilution computer (Vigilance, Edwards Lifesciences, Irvine, California, United States), by injecting 5 ml precooled saline in the pulmonary artery catheter. Systemic vascular (SV) and systemic vascular resistance (SVR) were calculated as: SV = CO/HR; SVR = (MAP – CVP) × 80/CO. DO_2_ and VO_2_ were calculated as O_2_ content in arterial blood × CO, and the difference of arterial and mixed venous O_2_ content × CO, respectively.

### Brain Microdialysis and Intracerebral Monitoring

An area of approximately 2 × 5 cm of the skull was exposed over the right hemisphere. A microdialysis catheter (CMA 70, CMA/Microdialysis, Stockholm, Sweden) was positioned at a depth of 10 mm below the duramater through a cranial hole positioned 1 cm right of the sagittal suture and 2 cm rostral to the coronal suture. The catheter was connected to a 1.0 ml syringe placed in a microinfusion pump (CMA102, CMA/Microdialysis) for perfusion of Ringer’s solution at a rate of 2.0 μL/min (Perfusion Fluid CNS, CMA/Microdialysis). Five samples were obtained during the experiment at 30 minutes intervals: at baseline at 30°C and 20°C during cooling, at 30°C during rewarming, and at 2 h after rewarming. The microvials containing the dialysate fluid were immediately frozen at –70°C and concentrations of cerebral tissue glucose, lactate, pyruvate, glutamate, and glycerol were measured using a microdialysis analyzer (CMA 600, CMA/Microdialysis).

A catheter for monitoring of intracerebral pressure (ICP) (Codman MicroSensor ICP Transducer, Codman & Shutleff, Raynham, Massachutes, United States/Millar transducer control unit TC-510, Houston, Texas, United States) and a temperature probe for monitoring intracerebral temperature were placed in the brain parenchyma just below the dura mater through a second cranial hole 1 cm to the right of the sagittal suture and 1 cm caudal to the coronal suture. ICP was displayed continuously on a monitor and recorded manually at different time intervals. Cerebral perfusion pressure (CCP) was calculated as MAP – ICP. Finally, a catheter was retrogradely placed through the left internal jugular vein to sample blood for determination of jugular venous O_2_ saturation.

### Use of Fluorescent Microspheres to Determine Regional Cerebral Blood Flow

#### Microsphere Injection

For measurements, 15 μm polystyrene fluorescent microspheres (FM), (FluoSpheres, Molecular Probes, Eugene, OR, United States) (Glenny, Bernard, and Brinkley 2585-97) of four different colors (orange, yellow-green, crimson, and red) were injected as follows; at baseline (38°C), at start and at the end of stable hypothermia (25°C), and after rewarming to (38°C). Before each injection, FM were resuspended using a Sonorex Digital 10P ultrasonic bath (Badelin electronic GmbH, Berlin, Germany) for 1 minute and vortexed for 1 minute before 1 ml of the FM suspension (1 × 10^6^ FM) was injected into the left ventricle through the 6F pigtail catheter followed by washing with 10 ml saline. Reference blood samples were drawn, using a withdrawal pump (Harvard Apparatus, Holliston, MA, United States) at a constant rate of 4.12 ml/min, into a preheparinized 20 ml syringe *via* a catheter inserted into the right femoral artery and advanced to the abdominal part of the aorta. The collection of the reference blood sample began 15 seconds before the FM injection and continued for 2 minutes. After sampling, the syringe and sampling line were rinsed twice with 5 ml saline and placed in the vial. After each experiment, brain biopsies were obtained from the frontal lobes, cerebellum (2–3 g), and hippocampus (0.5–1 g) were placed into sampling vials and processed later for regional blood flow analysis.

### Sample Processing

Tissue and blood samples were processed (digesting, filtration, rinsing, and dye extraction) using sample processing units (SPU) (Perkin-Elmer Analytical, Shelton, CT, United States) as described in detail by Raab et al. (1999). The SPU consists of three main parts: filter unit, filter holder, and sample tube with a screw-top cap. All tissue samples were weighed immediately after harvesting. To recover FM from tissue or blood, all samples were digested using 4 N aqueous KOH solution (224.4 g/l) with 2% Tween 80 (20 ml per 1,000 ml of KOH solution, provided by Sigma-Aldrich Norway AS, Oslo, Norway. Each tissue or reference blood sample was placed in a separate filter unit, which was then placed in high-grade steel beaker filled with 25 ml digesting solution, and 2.5 ml 100% isopropanol alcohol, covered with tight caps, and kept in a heating oven for 24 h at 60°C. After the digestion process, filter units were carefully removed from the steel beakers, and the liquid was suctioned through the wire mesh of the filter with a maximum negative pressure of 400 hPa. To neutralize residual KOH, the filter unit wall and the filter mesh were rinsed using 20 ml phosphate buffer (5.88 g KH_2_PO_4_ in 200 ml H_2_O + 29.9 g K_2_HPO_4_ in 800 ml H_2_O) before the filter units were immersed in a beaker filled with phosphate buffer to clean residual KOH from the outside. To remove remaining liquid from the filter units, they were placed in 50 ml plastic tubes and centrifuged for 3 minutes at 4,000 rpm. After digestion, suction, washing, and centrifuging, all FM recovered from tissue or blood were collected on a filter mesh inside the filter units. Then each filter unit was placed into the filter holder with the sample tube connected to the lower end of the filter holder. To extract dye from microspheres, 1 ml of organic solvent (Cellosolve, 2-ethoxyethyl acetate) was pipetted into the filter unit, and the assembly with filter unit inside was vortexed softly for 30 seconds. After 1 minute, a second extraction of dye using 1 ml of Cellosolve was performed. The assembly with filter unit inside was then centrifuged at 4,000 rpm for 30 seconds, and all Cellosolve- containing fluorescent dye samples were collected in a sample tube. The concentration of fluorescent dye dissolved in Cellosolve was measured using a microplate reader (Wallac Victor^2^_*TM*_ 1,420 Mulitlabel Counter, Perkin-Elmer Life Sciencis, Turku, Finland). Organ blood flow (OBF) was calculated based on the following formula:


OBF(ml/min/g)=(R×I)T/I×RWt.

Where, R is the withdrawal rate of the reference blood sample (4.12 ml/min), I_*T*_ is the fluorescence intensity of the tissue sample, I_*R*_ is the fluorescence intensity of the reference blood sample, and Wt is the weight of the tissue sample (g).

### Cooling and Rewarming Protocols

#### Temperature Monitoring

Body temperature was continuously monitored at four locations: brain (Licox temperature probe/Licox MCB Universal oxygen and temperature monitor, Mielkendorf, Germany); esophagus; urinary bladder (Kone temperature probes, Espoo, Finland); and pulmonary arterial blood (*via* the thermodilution catheter). During CPB, temperatures were also monitored in the venous and arterial lines and displayed on the centrifugal pump machine. Esophageal temperature was used as reference temperature for determining sampling points throughout experiments and the end point of cooling, whereas the point of weaning from CPB during rewarming was determined by urinary bladder temperature. At temperatures below 25°C, brain temperature was used to withdraw anesthetics.

#### Immersion Cooling (IMM_*c*_ Group Only)

Animals in the IMM_*c*_ group were placed in a right lateral recumbent position on the operating table. Using a centrifugal pump (Bio-Medicus, Eden Prairie, Minnesota, United States) and heat-exchanger (Stöckert Normo/hypothermie, Munich, Germany), cold water (5°C) was circulated in the hollow operating table and a tarpaulin tub surrounding the animal. The upper left side of the animal was covered with ice slush and irrigated with cold water, submerging 2/3 of the animals. The head was placed on a cushion and not immersed or covered with ice slush. As esophageal temperature fell below 24°C and serious bradycardia (< 20 beats/min) developed, circulation of cold water was discontinued, and the tub was drained of water and ice slush. Invariably, esophageal temperature subsequently dropped further, and with the onset of asystole preparations for sternotomy and attachment to CPB were started.

### Cardiopulmonary Bypass

#### Initiating and Maintaining Cardiopulmonary Bypass

A heparin-coated CPB circuit consisting of a membrane oxygenator (Jostra Quadrox, Maquet Cardiopulmonary, Hirrlingen, Germany), a hosing system without venous reservoir and a centrifugal pump head (Bio-Medicus, Eden Prairie, Minnesota, United States) was primed with approximately 600 ml of Ringer acetate. The heart was exposed *via* a median sternotomy. The ascending aorta was cannulated with a 16 F arterial cannula (Jostra), and a single stage 24 F venous cannula (Medtronic, Cardiac Surgical Products, Grand Rapids, Michigan United States) was placed in the right atrium. A 12 F intracardiac catheter was positioned into the left ventricle through the apex of the heart for decompression of the left ventricle. Blood coagulability was monitored using activated clotting time (ACT) (Hemochron 801, Hemochron whole blood coagulation system, Edison, New Jersey, United States). Heparin was given to keep ACT around 200 seconds. Non-pulsatile CPB was started, and pump flow adjusted to reach a global perfusion pressure above 50 mm Hg and a central venous saturation above 60%. If these endpoints were not reached by increasing the pump head rotation speed, Ringer acetate was added to the circuit. According to α-stat management, fresh gas administration was adjusted to maintain PaO_2_ supranormal and PaCO_2_ within normal limits.

#### Cooling and Rewarming by Cardiopulmonary Bypass

Cooling and rewarming while on CPB were performed using the heat-exchanger attached to the oxygenator to achieve a temperature gradient of 5°C maximum between drained venous blood and inflowing arterial blood. However, during rewarming maximum temperature in the inflowing arterial line was set at 39°C and the temperature gradient between the urinary bladder and blood in the arterial line was not allowed to exceed 10°C. To ensure the heart surgery resemblance; after cooling animals in the CPB_*c*_ group to18°C (in esophagus), CPB was stopped, the aorta was cross-clamped distal to the aortic cannula and a cold (4°C) crystalloid hyperkalemic cardioplegic solution (St. Thomas cardioplegic solution, 13 ml/kg) was added *via* the aortic cannula to produce cardiac arrest.

During rewarming internal electroconversion of ventricular fibrillation was initiated at an esophageal temperature of 25°C. If three attempts at electro-conversion were unsuccessful up to an esophageal temperature of 28°C, a bolus of 150 mg amiodarone was administered in the arterial cannula before additional electro-conversions.

Weaning from CPB was performed when the temperature measured in the urinary bladder reached 36°C, accomplished by use of dopamine (DA) infusions to keep MAP > 60 mmHg.

### Biochemical Analyses

Analyses of arterial, mixed venous, and jugular vein blood gases along with hemoglobin (Hb) measurements were performed using a blood gas analyzer (Rapid lab, Chiron Diagnostics, Emeryville, CA, United States) uncorrected for temperature. Blood samples for plasma analysis were put on ice, quickly centrifuged and frozen, and kept at –80°C before analysis. Troponin T (TnT), ASAT, ALAT, and albumin were analyzed using the sandwich method of electrochemical luminescence, UV-test with pyridoxal phosphate activation, and a colorimetric end point method (Modular, Roche Diagnostics, Rotkreuz, Switzerland).

### Postmortem Wet/Dry Organ Weight Ratios

A postmortem autopsy was performed, and representative samples of various organs were excised, weighed, and stored overnight in an incubator at 60°C, before weighing was repeated followed by calculation of wet/dry ratios.

### Statistical Analyses

Statistical analyses were performed using SigmaPlot statistical software version 11 (Systat Software Inc. (SSI), Richmond, California, United States). Intragroup comparisons were performed by one-way repeated measures analysis of variance, followed by *post hoc* pairwise comparisons using the Holm-Sidak or Student-Newman-Keuls tests when appropriate. For within group single comparisons a paired sample Student’s *t* test was used. For comparisons between groups, a two sample Student’s *t* test was used if data showed normal distribution, otherwise Mann-Whitney rank sum test was used. For comparisons of survival between groups the two-sided Barnard’s unconditional test was used. Level of significance was set at *p* ≤ 0.05. Data are presented as mean ± standard error of mean (SEM).

## Results

No statistically significant differences in any of the variables were observed in baseline measurements.

### Survival

Out of eight animals in the IMM_*c*_ group, four died at the time of weaning from CPB after rewarming was completed, or shortly thereafter. Death appeared to result from electro-mechanical dissociation since the heart had no measurable output in spite of electrical activity. In contrast, all animals in the CPB_*c*_ group survived for the 2-hour period post CPB. Thus, intergroup difference in survival was statistically significant (*p* = 0.02).

### Flow, Pressures, and O_2_ Variables

#### Cooling

**FIGURE 3:**
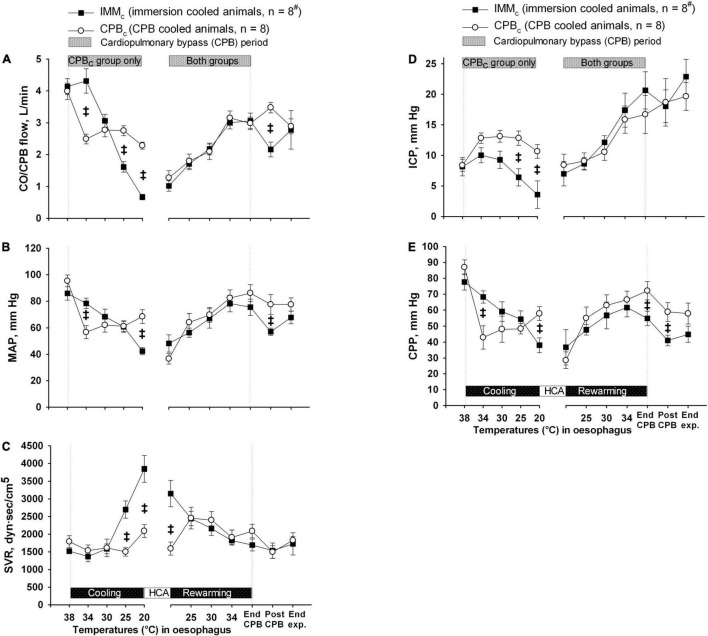
Blood flow and pressures. **(A)** Cardiac output and CPB flow (CO/CPB flow). **(B)** Mean arterial pressure (MAP). **(C)** Systemic vascular resistance (SVR). **(D)** Intracranial pressure (ICP). **(E)** Cerebral perfusion pressure (CPP). Data presented as mean and SEM. ^‡^Statistically significant difference between groups (*p* < 0.05). ^#^n was reduced to 5 animals after termination of CPB (Post CPB) and to 4 animals at end of experiments (End exp.) in IMM_*c*_ group.

**FIGURE 4:**
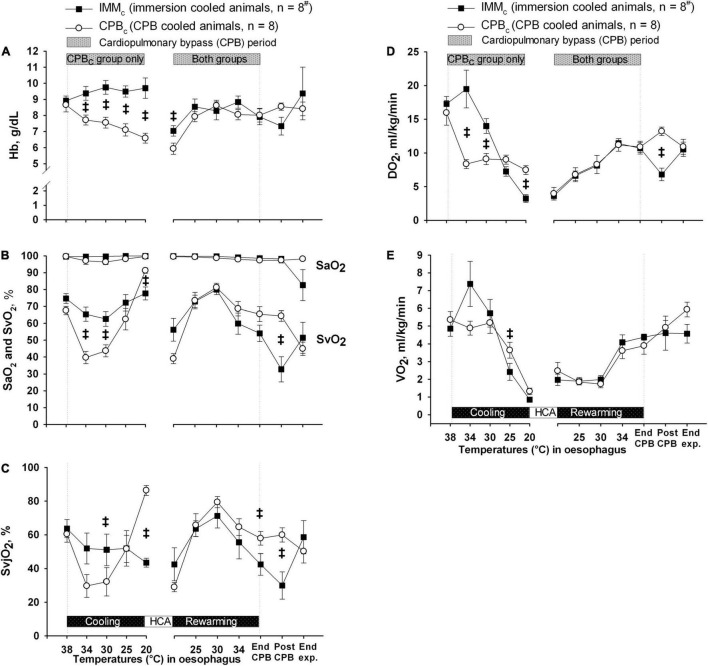
Oxygen variables. **(A)** Blood hemoglobin (Hb) concentration. **(B)** Arterial and mixed venous blood oxygen saturation (SaO_2_ and SVO_2_). **(C)** Internal jugular venous blood oxygen saturation (SvjO_2_). **(D)** Global delivery of oxygen (DO_2_). **(E)** Global consumption of oxygen (VO_2_). Data presented as mean and SEM. ^‡^Statistically significant difference between groups (*p* < 0.05). ^#^n was reduced to 5 animals after termination of CPB (Post CPB) and to 4 animals at end of experiments (End exp.) in IMM_*c*_ group.

Except from the initial reduction in global blood flow delivered by the extracorporeal circuit compared with the flow generated by spontaneous circulation, animals in CPB_*c*_ group had a significantly higher blood flow below 34°C than IMM_*c*_ animals (Figure 3A). However, as Hb content was significantly lower in the CPB_*c*_ group after initiating CPB, and throughout cooling (Figure 4A), DO_2_ (Figure 4D) was significantly higher in IMM_*c*_ animals until 25°C. Below 25°C, CPB_*c*_ animals had significantly higher DO_2_ than IMM_*c*_ animals. Global extraction of O_2_, measured by SvO_2_ (Figure 4B), matched variations in DO_2_ so that VO_2_ (Figure 4E) was independent of DO_2_. Brain O_2_ extraction, measured by SvjO_2_, followed the pattern of changes in SvO_2_, but the difference between groups was more profound for SvjO_2_ than for SvO_2_ at 20°C (Figure 4C).

Mean arterial pressure (Figure 3B) declined steadily in IMM_*c*_ group, while SVR (Figure 3C) increased throughout cooling. In CPB_*c*_ group MAP stabilized after an initial drop at the onset of CPB, while SVR was unaffected by cooling. Consequently, at 20°C MAP was significantly lower in IMM_*c*_ group than in CPB_*c*_ group, while the opposite was the case for SVR.

During cooling, ICP declined in IMM_*c*_ group, while it was stable in CPB_*c*_ group, resulting in significantly higher values in CPB_*c*_ group at severe hypothermia (Figure 3D). However, with the decline in MAP in the IMM_*c*_ group, CPP in this group was significantly lower than in the CPB_*c*_ group at 20°C (Figure 3E).

*Rewarming.* Except for the initial increase in SVR and Hb in the IMMc group immediately after the start of rewarming by CPB, there were no intergroup differences in CPB flow, MAP, SVR, ICP, CPP, or O_2_ variables during rewarming. However, after completion of rewarming, just before weaning from CPB, CPP and SvjO were significantly higher in the CPB_*c*_ group.

*Post-hypothermic period:* In order to preserve statistical power, comparisons of variables between the groups after rewarming were made immediately after weaning from CPB, when five animals in the IMM_*c*_ group were still alive (Table 1). At this timepoint, animals in the IMM_*c*_ group had significantly lower CO, HR, MAP, CPP, SvO_2_, SvjO_2_, and DO_2_ than CPB-cooled animals (Table 1). SV did not differ in between the groups. Animals in the CPB_*c*_ group maintained CO at prehypothermic levels by increasing HR (Table 1). Further, CVP and average DA dose (7.4 ± 1.6 μg/kg/min in the IMM_*c*_ group vs. 2.1 ± 1.2 μg/kg/min in the CPB_*c*_ group) to support circulation were both significantly higher in IMM_*c*_ animals (Table 1).

**TABLE 1:** Flow- and pressure-derived variables, oxygen variables and dopamine administration before and after cooling.

Variable	Group	Baseline values	Post rewarming
**CO** (L/min)	**IMM_*c*_**	4.1 ± 0.2	2.2 ± 0.2***/+ + +
	**CPB_*c*_**	4.0 ± 0.3	3.5 ± 0.2
**HR** (beats/min)	**IMM_*c*_**	100 ± 3	106 ± 11 **+**
	**CPB_*c*_**	97 ± 7	137 ± 7**
**SV** (ml)	**IMM_*c*_**	41.3 ± 1.4	21.4 ± 3.6***
	**CPB_*c*_**	41.8 ± 3.0	25.7 ± 1.6**
**MAP** (mm Hg)	**IMM_*c*_**	85.9 ± 5.0	57.2 ± 3.3***/+
	**CPB_*c*_**	95.3 ± 4.5	77.5 ± 7.6
**ICP** (mm Hg)	**IMM_*c*_**	8.16 ± 1.51	18.07 ± 3.47*
	**CPB_*c*_**	8.37 ± 1.0	18.69 ± 3.88*
**CPP** (mm Hg)	**IMM_*c*_**	77.7 ± 5.1	41.2 ± 5.3***/+
	**CPB_*c*_**	87.0 ± 4.7	58.9 ± 5.8*
**SVR** (dyn⋅sec/cm^5^)	**IMM_*c*_**	1520 ± 69	1535 ± 216
	**CPB_*c*_**	1791 ± 169	1494 ± 180
**PVR** (dyn⋅sec/cm^5^)	**IMM_*c*_**	141 ± 25	414 ± 82*
	**CPB_*c*_**	179 ± 31	408 ± 65*
**CVP** (mm Hg)	**IMM_*c*_**	7.8 ± 1.0	18.0 ± 1.4***/+ +
	**CPB_*c*_**	9.3 ± 1.0	13.0 ± 0.8**
**dP/dt_*max*_** (mmHg/sec)	**IMM_*c*_**	2898 ± 220	2288 ± 310**
	**CPB_*c*_**	2656 ± 213	2655 ± 374
**dP/dt_*min*_** (mmHg/sec)	**IMM_*c*_**	–2492 ± 210	–1376 ± 202**
	**CPB_*c*_**	–2577 ± 191	–1910 ± 241
**LVEDP** (mm Hg)	**IMM_*c*_**	12.8 ± 2.0	17.7 ± 2.2*
	**CPB_*c*_**	15.6 ± 1.6	17.2 ± 2.4
**Hb** (g/dl)	**IMM_*c*_**	8.9 ± 0.3	7.3 ± 0.7
	**CPB_*c*_**	8.7 ± 0.4	8.6 ± 0.2
**SaO_2_** (%)	**IMM_*c*_**	99.2 ± 0.2	82.6 ± 9.2
	**CPB_*c*_**	99.6 ± 0.2	98.2 ± 1.1
**SvO_2_** (%)	**IMM_*c*_**	74.7 ± 2.9	32.8 ± 7.4**/+ + +
	**CPB_*c*_**	67.6 ± 2.4	64.3 ± 3.2
**SvjO_2_** (%)	**IMM_*c*_**	63.7 ± 5.4	30.0 ± 9.6*/+ +
	**CPB_*c*_**	60.4 ± 4.8	59.9 ± 4.2
**DO_2_** (mlO_2_/kg/min)	**IMM_*c*_**	17.3 ± 1.1	6.8 ± 0.9***/+ + +
	**CPB_*c*_**	15.9 ± 1.8	13.2 ± 0.6
**VO_2_** (mlO_2_/kg/min)	**IMM_*c*_**	4.9 ± 0.4	4.6 ± 1.0
	**CPB_*c*_**	5.4 ± 0.5	4.9 ± 0.4
**Dopamine** (μg/kg/min)	**IMM_*c*_**	0	7.4 ± 1.6 **+**
	**CPB_*c*_**	0	2.1 ± 1.2

IMM_c_, immersion cooled animals; CPB_c_, animals cooled by cardiopulmonary bypass; CO, cardiac output; HR, heart rate; SV, stroke volume; MAP, mean arterial pressure; ICP, intracranial pressure; CPP, cerebral perfusion pressure; SVR, systemic vascular resistance; PVR, pulmonary vascular resistance; CVP, central venous pressure; dP/dt_max_, maximal acceleration of pressure in the cardiac cycle; dP/dt_min_, maximal deceleration of pressure in the cardiac cycle; LVEDP, left ventricular end diastolic pressure; Hb, hemoglobin concentration; SaO_2_, hemoglobin oxygen saturation in arterial blood; SvO_2_, hemoglobin oxygen saturation in mixed venous blood; SvjO_2_, hemoglobin oxygen saturation in internal jugular vein blood; DO_2_, global delivery of oxygen; VO_2_, global consumption of oxygen. *p < 0.05 within groups from baseline; **p < 0.01 within groups from baseline; ***p < 0.001 within groups from baseline. + p < 0.05 between groups; + +p < 0.01 between groups; + ++p < 0.001 between groups.

### Brain Metabolism

**FIGURE 5:**
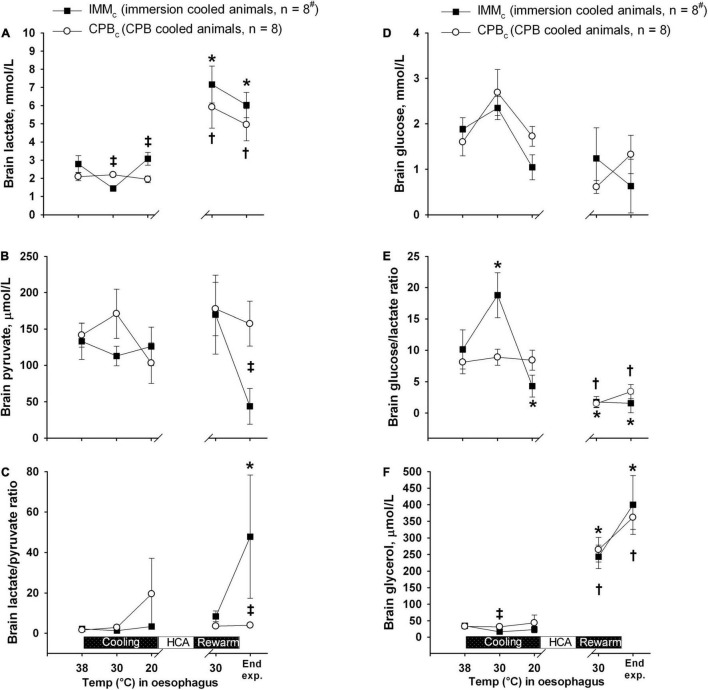
Brain microdialysate analyses. **(A)** Brain lactate. **(B)** Brain pyruvate. **(C)** Brain lactate/pyruvate ratio. **(D)** Brain glucose. **(E)** Brain glucose/lactate ratio. **(F)** Brain glycerol. Data presented as mean and SEM. *Statistically significant difference from baseline within IMM_*c*_ group, and ^†^within CPB_*c*_ group (*p* < 0.05). ^‡^ Statistically significant difference between groups (*p* < 0.05). ^#^n was reduced to 5 animals after termination of CPB (Post CPB) and to 4 animals at end of experiments (End experiment) in IMM_*c*_ group.

Brain lactate and glycerol were significantly increased in both the groups at the end of experiment, while the brain glucose/lactate ratio was decreased (Figure 5). At end of experiment, brain pyruvate was significantly lower in the IMM_*c*_ than in the CPB_*c*_ group, while the brain lactate/pyruvate ratio was significantly depressed in the IMM_*c*_ group compared to CPB_*c*_ animals.

### Regional Cerebral Blood Flow

**FIGURE 6:**
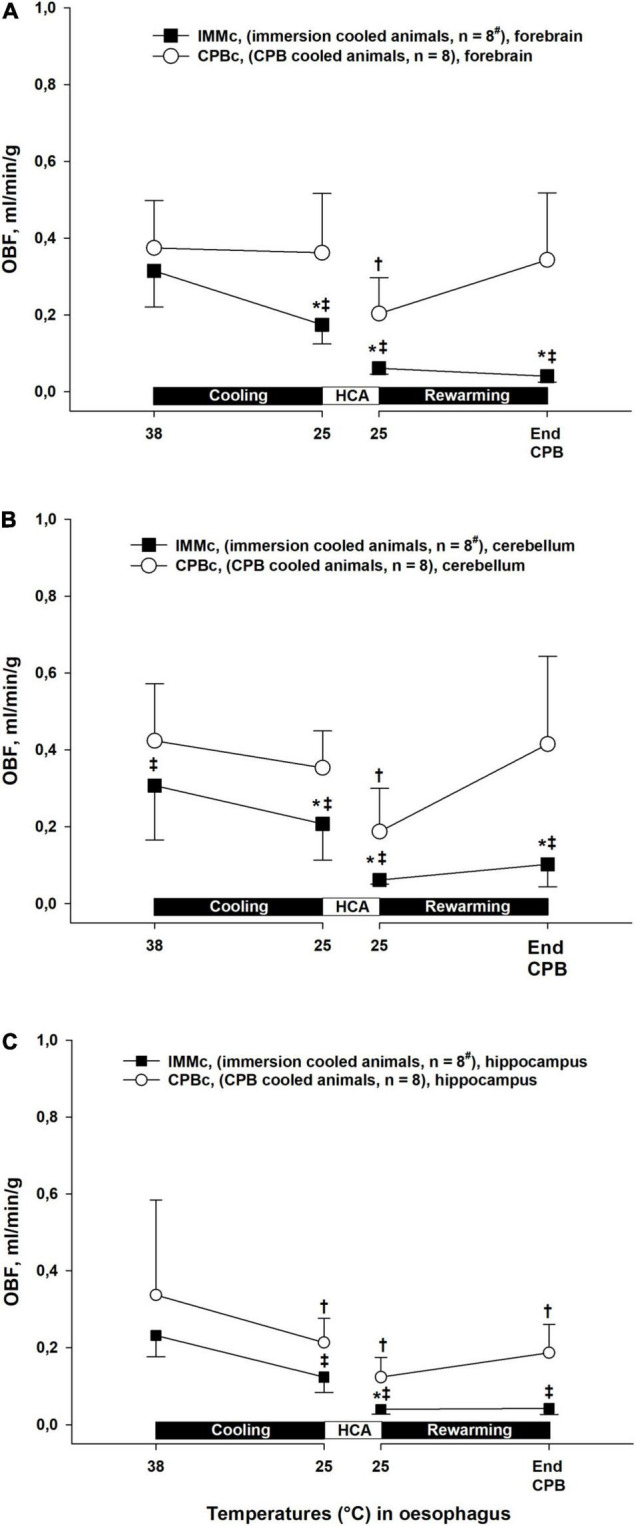
Cerebral blood flow. **(A)**: forebrain, **(B)**: cerebellum, **(C)**: hippocampus. Data presented as mean and SEM. *Statistically significant difference from baseline within IMM_*c*_ group, and ^†^within CPB_*c*_ group (*p* < 0.05). ^‡^ Statistically significant difference between groups (*p* < 0.05). ^#^n was reduced to 5 animals after termination of CPB (Post CPB) and to 4 animals at end of experiments (End exp.) in IMM_*c*_ group.

During cooling and rewarming, blood flow in forebrain, hippocampus, and cerebellum were higher in the CPB_*c*_ group as compared to IMM_*c*_ animals (Figure 6). Further, in the CPB_*c*_ group, blood flow in forebrain and cerebellum was significantly reduced at 25°C during rewarming compared to baseline (38°C), whereas blood flow in hippocampus remained reduced after cooling to 25°C. In the IMM_*c*_ group, blood flow in forebrain and cerebellum remained reduced compared to 38°C baseline values throughout the cooling and rewarming protocol, whereas blood flow in hippocampus was reduced only at 25°C rewarming.

### Biochemical Variables

**FIGURE 7:**
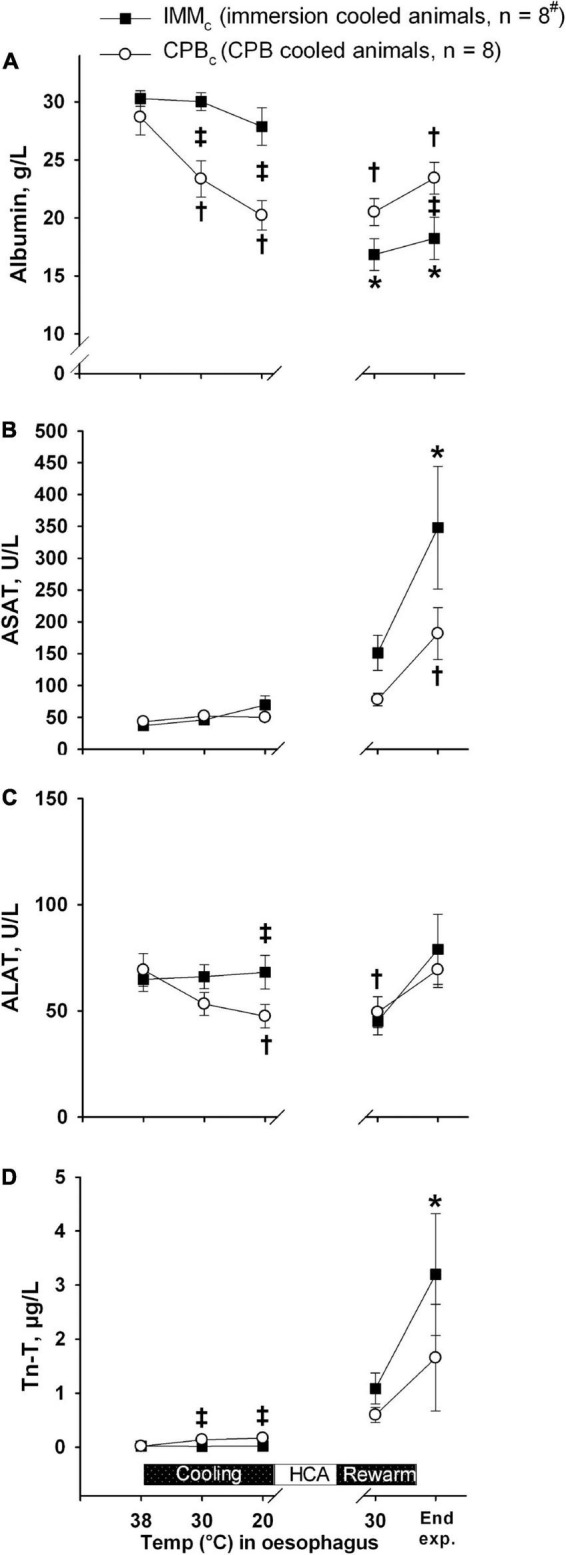
Biochemical analyses. **(A)**: Albumin; **(B)**: ASAT; **(C)**: ALAT; **(D)**: Troponin-T (Tn-T). Data presented as mean and SEM. *Statistically significant difference from baseline within IMM_*c*_ group, and ^†^within CPB_*c*_ group (*p* < 0.05). ^‡^Statistically significant difference between groups (*p* < 0.05).

Plasma albumin values decreased significantly in the CPB_*c*_ group after initiating CPB during cooling, compared to both their own baseline values and IMM_*c*_ animals (Figure 7). However, this difference in plasma albumin between groups was reversed during and after rewarming, with significantly higher albumin values in CPB_*c*_ group by the end of experiment. A this time point, this was the only significant intergroup difference in biochemical variables.

### Fluid Administration Rates Weight Gains and Organ Weight Wet/Dry Ratios

**FIGURE 8:**
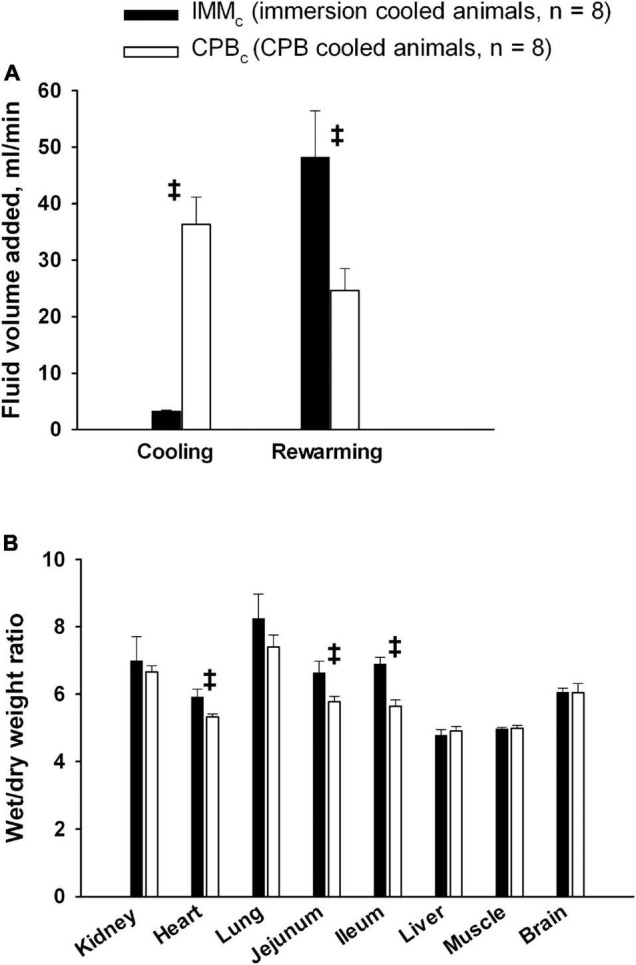
Fluid administration and organ edema. **(A)**: Fluid volume administration rate. **(B)**: Postmortem wet/dry organ weight ratio. Data presented as mean and SEM. ^‡^Statistically significant difference between groups (*p* < 0.05).

The administration rate of fluid volume was significantly higher during cooling by CPB than by immersion cooling (Figure 8A). However, fluid administration rate in IMM_*c*_ animals was significantly higher in the CPB_*c*_ group during rewarming by CPB. However, no differences between groups in overall fluid administration rates and total body weight gain ratios were found. Post-mortem wet/dry organ weight ratios (Figure 8B) showed significantly increased water content in the heart and gut in the IMM_*c*_ group compared to the CPB_*c*_ group.

### Cooling and Rewarming Times and Temperature Measurements

The total experiment times did not differ significantly between the groups (10 h, 28 ± 7 min in IMM_*c*_ and 10 h, 11 ± 8 min in CPB_*c*_ group (Figure 2). As expected, cooling was significantly slower in IMM_*c*_ group (1 h 52 ± 10 min) compared to the CPB_*c*_ group (52.8 ± 2.8 min, *p* < 0.001). But as considerable time was used for surgical preparation in the latter group, there was no significant time difference between groups in reaching cooling end-points, measured from baseline. Rewarming lasted significantly longer in the IMM_*c*_ group (1 h, 19 ± 7 min) compared to in the CPB_*c*_ group (56 ± 3 min, *p* = 0.007). Total CPB time was longer in the CPB_*c*_ group (1 h 48 ± 5 min) when compared to rewarming time only in IMM_*c*_ animals (*p* = 0.005). Figure 1A shows that temperatures in different organs (esophagus, urinary bladder, pulmonary artery, and brain) were relatively consistent in the IMM_*c*_ group during cooling, while temperature in the urinary bladder lagged behind during the much more efficient extracorporeal cooling in the CPB_*c*_ group (Figure 1B). Surviving IMM_*c*_ animals did not manage to preserve body heat after weaning from CPB, resulting in significantly lower temperatures than in the CPB_*c*_ group at end of experiments (Figures 2A,B).

## Discussion

The results of the present study show that after rewarming, IMM_*c*_ animals suffered from severe cardiovascular failure at weaning from CPB, leading to imminent or premature death in 4 out of 8 animals. In contrast, all animals in the CPB_*c*_ group survived the 2-hour post CPB rewarming period. This finding underlines the well-recognized differences in success rate from CPB rewarming in accidental hypothermia compared to conventional CPB rewarming after cardiac surgery.

Animals in both groups were subjected to 75 minutes of global hypothermic ischemia, but post-hypothermic perturbation of cardiovascular function was far more severe in IMM_*c*_ animals. The underlying pathophysiology may reflect hypothermic and/or ischemic damage during immersion cooling prior to HCA. In the pioneering work of Bigelow and colleagues, a significant reduction in CO was reported in dogs upon rewarming after cooling to HCA (Bigelow et al., 1950). These results were consistent with other studies showing cardiac dysfunction upon rewarming after cooling but without cardiac arrest (Prec et al., 1949; Ross, 1954; Blair et al., 1956; Fedor et al., 1958; Popovic and Kent, 1965; Steen et al., 1980; Tveita et al., 1994, 1996). The introduction of CPB and cardioplegic agents to achieve diastolic cardiac arrest before cardiac surgery (also applied during HCA in the actual CPB_*c*_ group) seemed to alleviate post-hypothermic cardiac dysfunction (Taufic and Lewis, 1953). The pathophysiology of post-operative cardiac dysfunction is considered to be related to the preceding period of ischemia-reperfusion during surgery and not to the hypothermic exposure *per se* (Toller and Metzler, 2005). However, post-hypothermic cardiac dysfunction remains a clinical challenge related to rewarming from accidental hypothermia.

Inspired by these original research works (Prec et al., 1949; Bigelow et al., 1950, 1954; Fairley et al., 1957) our group has conducted experimental hypothermia/rewarming studies by use of intact animal models (Tveita et al., 1991a,b, 1993, 1994, 1996, 1998a,b, 1999) and *in vitro* heart muscle tissue (Han et al., 2010, 2018). Results from our studies are consistent with their findings of reduced myocardial function after rewarming (Filseth et al., 2010, 2012). Our results have consistently revealed that the hypothermia and rewarming cause direct effects on myocardial excitation-contraction coupling and actin-myosin interaction (Tveita et al., 1998b; Han et al., 2008; Filseth et al., 2010). These effects are most likely to explain the depression in left ventricular contractile function after rewarming, which underlies the reduction of cardiac output and systemic arterial pressure (Tveita, 2000). Impaired Ca^2+^ control is a key factor in the pathophysiology of heart failure in normothermia (Vassalle and Lin, 2004). Also, hypothermia/rewarming appears to disturb the mechanisms underlying excitation-contraction coupling in cardiac muscle force generation, and over time Ca^2+^ overload occurs (Tani and Neely, 1989; Vassalle and Lin, 2004), which results in mechanical dysfunction that may entail acute cardiac dysfunction. However, during rewarming in isolated cardiac myocytes the amplitude and duration of the evoked [Ca^2+^]_*cyt*_ transient returns to normothermic levels (Schaible et al., 2016). Thus, it does not appear that the decrease in contractility of cardiac myocytes (heart failure) after rewarming can be solely attributed to dysregulation of [Ca^2+^]_*cyt*_ release and reuptake. By the use of isolated, electrically stimulated myocytes cooled to 15°C, and rewarmed we have documented that the reduction in force generation, independent of [Ca^2+^]_*cyt*_ levels, is the effect of an increased phosphorylation of cardiac troponin I (cTnI) leading to reduced Ca^2+^ sensitivity of the contractile response after rewarming (Han et al., 2010; Schaible et al., 2016, 2018). Further, with relevance to the group (CPBc) treated with a chemical cardioplegic solution during HCA in the present study; we observed both maintained cytosolic Ca^2+^ levels and myofilament Ca^2+^ sensitivity in myocytes, which were not electrically stimulated (cardioplegic) during cooling to 15°C and rewarming (Han et al., 2018).

The increase in O_2_ extraction in the IMM_*c*_ versus the CPB_*c*_ group compensated for the lower CO in this group and made global VO_2_ similar in the two groups. However, in IMM_*c*_ animals below 25°C, the more than doubled SVR, combined with reduced MAP, may have contributed to a compromised regional tissue perfusion.

It was also noted that animals in the IMM_*c*_ group that could not be weaned from CPB had a contracted, non-beating heart, similar to a “stone heart”. Before cardioplegic solutions and hypothermic bypass were introduced during the 1970’s, this clinical phenomenon, later linked to cytosolic Ca^2+^ accumulation (Jimenez et al., 1990), was a feared complication to ischemic cardiac arrest during heart surgery (Cooley et al., 1972). In the present study, the irreversible contracture developed in half of the IMM_*c*_ animals during rewarming. If one should translate to the clinical situation, patients displaying a severe post-hypothermic cardiovascular failure should be supported by prolonged ECMO. Based on promising clinical reports (Ruttmann et al., 2007) the use of ECMO for rewarming from accidental hypothermia has been recommended as ECMO can also be continued after rewarming for cardio/respiratory support for days, if needed (Ruttmann et al., 2007; Morita et al., 2011). Recently, we have demonstrated that by the use of the present model that rewarming with ECMO restored blood flow to the heart and brain and created a “shockable” cardiac rhythm after 3 h of continuous CPR at 27°C (Nilsen et al., 2021).

It has been documented that a MAP as low as 40 mmHg during hypothermic CPB may lead to cerebral ischemia, indicated, among other variables, by increased levels of lactate and lactate/pyruvate ratio in brain microdialysate fluid (Haugen et al., 2006). Our finding of significantly increased brain lactate in IMM_*c*_ animals compared to CPB_*c*_ animals at the onset of HCA was preceded by significant drops in both CPP and SvjO_2_. Together with a significantly increased post-hypothermic brain lactate/pyruvate ratio in IMM_*c*_ compared to CPB_*c*_ animals, this may indicate that the brain suffered ischemic injury during spontaneous circulation below 25°C. The last conclusion finds support in the cerebral blood flow data in IMM_*c*_ animals versus CBF_*c*_ at 25°C showing significantly lower flow in IMM_*c*_ in all three parts of the brain investigated: forebrain, hippocampus, and cerebellum. These differences in cerebral blood flow between groups remained throughout the intervention protocol, and possible mechanisms to explain these differences in blood flow may be found in other preclinical studies conducted during the early attempts to establish transplantation medicine. They demonstrated that the application of cold *per se*, by simply cooling an organ, is not sufficient to provide good preservation of donor organs. Follow up studies showed that hypothermia was associated with an increase in blood viscosity that contributes to red cell sludging in the microvasculature as well as detrimental coagulopathies (Dewall et al., 1962; Johansson and Huttunen, 1963; Suzuki and Penn, 1965; Michenfelder and Theye, 1970; Baumgartner et al., 1983). Other experiments showed increased survival during profound hypothermic CPB combined with exsanguination and substitution with acellular solutions, and successful rewarming with auto-transfusion after prolonged hypothermia (Taylor et al., 1995).

Further, extravasation of plasma fluid has been documented to take place during normothermic CPB, but this extravasation increases significantly during hypothermic CPB (Heltne et al., 2001; Farstad et al., 2003). Extravasation may also occur in surface cooling with maintained circulation, but obviously to a lesser extent (Hammersborg et al., 2005). *Per protocol* more fluid volume was added to the extracorporeal circuit in the IMMc group than in the CPB_*c*_ group during rewarming (Figure 3A). Therefore, one could speculate that capillary leak was greater during CPB rewarming in IMM_*c*_ animals.

## Limitations

The combined hypothermic and ischemic injury inflicted to the animals in the IMM_*c*_ group that resulted in immediate or delayed death in 4 out of 8 animals after rewarming and before the end of experiment, clearly affected power of post-hypothermic statistics. Furthermore, because only the fittest animals in IMMc group survived to the end, results in the post-hypothermic period may have been biased.

## Conclusion

The translational value of this experiment is in order to create successful CPB-rewarming with favorable neurologic outcome in victims of accidental hypothermia, the CPB strategies need to differ from those applied in conventional cardiac surgery. Above all, the need for prolonged ECMO should be considered in all victims of HCA that cannot be weaned from CPB immediately after rewarming.

## Data Availability Statement

The raw data supporting the conclusions of this article will be made available by the authors, without undue reservation.

## Ethics Statement

The animal study was reviewed and approved by the Norwegian Animal Research Authority.

## Author Contributions

OF conducted the experiments and, together with TT and GS, designed the protocol, discussed the data, and wrote the manuscript. SH took part in carrying out experiments and contributed to the writing process. TK took part in carrying out experiments and processing and interpretation of data. All authors contributed to the article and approved the submitted version.

## Conflict of Interest

The authors declare that the research was conducted in the absence of any commercial or financial relationships that could be construed as a potential conflict of interest.

## Publisher’s Note

All claims expressed in this article are solely those of the authors and do not necessarily represent those of their affiliated organizations, or those of the publisher, the editors and the reviewers. Any product that may be evaluated in this article, or claim that may be made by its manufacturer, is not guaranteed or endorsed by the publisher.
